# Structural insights into the differences among lactisole derivatives in inhibitory mechanisms against the human sweet taste receptor

**DOI:** 10.1371/journal.pone.0213552

**Published:** 2019-03-18

**Authors:** Tomoya Nakagita, Akiko Ishida, Takumi Matsuya, Takuya Kobayashi, Masataka Narukawa, Takatsugu Hirokawa, Makoto Hashimoto, Takumi Misaka

**Affiliations:** 1 Department of Applied Biological Chemistry, Graduate School of Agricultural and Life Sciences, The University of Tokyo, Tokyo, Japan; 2 Department of Cell Biology, Graduate School of Medicine, Kyoto University, Kyoto, Japan; 3 Graduate School of Agriculture, Hokkaido University, Sapporo, Japan; 4 Molecular Profiling Research Center for Drug Discovery, National Institutes of Advanced Industrial Science and Technology, Tokyo, Japan; 5 Department of Chemical Biology, Faculty of Medicine, University of Tsukuba, Ibaraki, Japan; Leibniz-Institute for Food Systems Biology at the TU Munich, GERMANY

## Abstract

Lactisole, an inhibitor of the human sweet taste receptor, has a 2-phenoxypropionic acid skeleton and has been shown to interact with the transmembrane domain of the T1R3 subunit (T1R3-TMD) of the receptor. Another inhibitor, 2,4-DP, which shares the same molecular skeleton as lactisole, was confirmed to be approximately 10-fold more potent in its inhibitory activity than lactisole; however the structural basis of their inhibitory mechanisms against the receptor remains to be elucidated. Crystal structures of the TMD of metabotropic glutamate receptors, which along with T1Rs are categorized as class C G-protein coupled receptors, have recently been reported and made it possible to create an accurate structural model for T1R3-TMD. In this study, the detailed structural mechanism underlying sweet taste inhibition was characterized by comparing the action of lactisole on T1R3-TMD with that of 2,4-DP. We first performed a series of experiments using cultured cells expressing the sweet taste receptor with mutations and examined the interactions with these inhibitors. Based on the results, we next performed docking simulations and then applied molecular dynamics-based energy minimization. Our analyses clearly revealed that the (*S*)-isomers of both lactisole and 2,4-DP, interacted with the same seven residues in T1R3-TMD and that the inhibitory potencies of those inhibitors were mainly due to stabilizing interactions mediated via their carboxyl groups in the vertical dimension of the ligand pocket of T1R3-TMD. In addition, 2,4-DP engaged in a hydrophobic interaction mediated by its *o*-Cl group, and this interaction may be chiefly responsible for the higher inhibitory potency of 2,4-DP.

## Introduction

In humans, the taste receptor type 1 family (T1Rs), which are classified as class C G-protein coupled receptors (GPCRs), make a major contribution to the recognition of major nutrients such as sugars and amino acids, which represent major energy sources in human diets, in our oral cavity [1]. The sugar-recognizing sweet taste receptor, which consists of a heterodimer of T1R2 and T1R3 [2–6], has been shown to contain multiple ligand-binding sites in its structure [7]. Low-molecular-weight sweeteners, such as sugar, sucralose, aspartame, saccharin, and acesulfame K, are recognized within the extracellular Venus flytrap domain of T1R2 (T1R2-VFTD), which is considered to be an orthosteric binding site [8,9]. In our previous study, in which we sought to elucidate the mode of action between the human sweet taste receptor and those individual sweeteners, we established cell lines that stably express each of the point mutants for the sweet taste receptor together with a chimeric G-protein (Gα16gust44) and performed a series of mutational analyses that successfully revealed suitable candidates for residues in T1R2-VFTD that interact with such sweeteners [9].

Another binding site in the sweet taste receptor was found in the transmembrane domain of T1R3 (T1R3-TMD). This domain has been shown to be characterized by features that enable its interaction with both sweeteners and sweet taste inhibitors [10–13]. It has been shown that sweeteners such as cyclamate and neohesperidin dihydrochalcone (NHDC) both interact with T1R3-TMD, revealing that they are recognized as sweet by themselves (in other words, they actually act as agonists), and act as positive allosteric modulators (PAMs) of orthosteric ligands; they are therefore termed ago-PAMs [14–18].

Sweet taste inhibitors, such as lactisole, also interact with T1R3-TMD but act as negative allosteric modulators (NAMs). Lactisole was originally isolated from roasted coffee beans [19]. Because the molecule contains a lactic acid structure, lactisole has naturally occurring optical isomers. In general, there is more L-lactic acid [(*S*)-lactic acid] than D-lactic acid in plants, so the (*S*)-isomer of lactisole is predominant in roasted coffee beans [20]. Several other inhibitors of sweet taste receptors have also been characterized in previous publications. Another sweet taste inhibitor, 2,4-DP (2-(2,4-dichlorophenoxy)propionic acid), has a 2-phenoxypropionic acid skeleton similar to that of lactisole [21]. Although 2,4-DP also interacts with T1R3-TMD and exhibits approximately 10-fold higher inhibitory potency than lactisole, the structural basis for this difference in potency has not been elucidated.

In addition, a previous publication proposed an interaction model between T1R3-TMD and lactisole based on the crystal structure of rhodopsin, a representative class A GPCR [11]. However, the amino acid sequence identity between rhodopsin and T1R3-TMD is quite low, making it unlikely that the constructed structural model for T1R3-TMD accurately describes the detailed structural mechanisms of lactisole-T1R3 interactions. Recently, the crystal structures of the transmembrane domain of metabotropic glutamate receptors (mGluRs), which along with T1Rs are categorized as class C GPCRs, were solved [22,23]. These structures made it possible to create an accurate interaction model of T1R3-TMD.

In this study, we sought to characterize the similarities and differences between the modes of action of lactisole and 2,4-DP by creating docking models with T1R3-TMD. We reasoned that common features are likely to be important for inhibitory activity, and distinct features are likely to be responsible for differences in inhibitory potency among those inhibitors.

## Materials and methods

### Cell-based assay

#### Construction of cell lines stably expressing the sweet taste receptor

The previously reported method to construct a cell line stably expressing the human sweet taste receptor [14,24] was also used in this study. The genes encoding T1R2, T1R3, and Gα16gust44 were incorporated into a modified version of the pcDNA5/FRT vector (Thermo Fisher Scientific, Waltham, MA, U.S.A.). Following the Flp-In System protocol (Thermo Fisher Scientific), this construct was transfected into Flp-In 293 cells. Each cell line stably expressing a variant of the sweet taste receptor was generated in the same manner. Nucleotide mutations were introduced into the expression construct by PCR-based mutations. The residues to be mutated were primarily chosen based on previous studies [10–12].

#### Measurement of cellular responses

Measurements of cellular responses were performed as described previously [14,24]. Cells stably expressing the sweet taste receptor were seeded in 96-well plates and incubated for an additional 23 hours. The cells were washed with assay buffer and loaded using the FRIPR Calcium 4 Assay Kit (Molecular Devices, San Jose, CA, U.S.A.). Measurement was performed on FlexStation 3 (Molecular Devices) after the samples were incubated at 37 °C for 1 hour. The temperature of the FlexStation 3 was also maintained at 37 °C.

#### Data analysis

All data were fitted to Hill’s equation, which was drawn using Clampfit 9.2 (Molecular Devices, Palo Alto, CA, USA), and EC_50_ and IC_50_ values were calculated from a dose-response curve.

### Computer simulation

#### Creation of the homology model of T1R3-TMD

All calculations were conducted using Schrödinger Suite 2017–1 (Schrödinger, LLC) and performed under the OPLS3 force field. First, the crystal structure of mGluR1-TMD (Protein Data Bank ID code: 4OR2) was prepared, then the T1R3-TMD homology model was created with reference to alignments of mGluR1, mGluR5, and T1Rs. The result of alignment is shown in S1 Fig (according to GPCRdb (http://gpcrdb.org), and the sequence identities vs T1R3 were mGluR1, 20%; mGluR5, 18%; T1R1, 27%; and T1R2, 22% respectively. (As a side note, sequence identities vs class A GPCRs were quite low: rhodopsin, 9% and β2-adrenoceptor, 6%, respectively. (S1B Fig)). The alignments and calculation to create the model were performed with Prime (Schrödinger). Insertion gaps were considered using both structures of mGluR1 (4OR2) and mGluR5 (4OO9). All residues shown in this study are labeled with superscript characters according to generic GPCR residue numbers [25].

#### Ligand docking

Five ligands, (*S*)-lactisole, (*R*)-lactisole, (*S*)-2,4-DP, (*R*)-2,4-DP, and (*S*)-2-phenoxypropionic acid were calculated in advance, with proper form and ionic charge, using LigPrep (Schrödinger). The ligands were then docked to the ligand pocket surrounded by residues identified by mutational analysis. Docking simulations were performed using Glide (Schrödinger) in Standard Precision-mode. After docking, we recalculated the ligand pocket configuration using the Protein Preparation function in Maestro.

#### Molecular dynamics-based energy minimization

(*S*)-lactisole, (*R*)-lactisole, (*S*)-2,4-DP, and (*R*)-2,4-DP were subjected to four independent molecular dynamics (MD) simulations using Desmond (Schrödinger). The initial docking models were placed into a large POPC bilayer, and TIP3P water molecules were solvated with 0.15 M NaCl. After minimization and relaxation of the model, the production MD phase was performed for 100 ns in the isothermal-isobaric (NPT) ensemble at 300 K and 1 bar using a Langevin thermostat. Long-range electrostatic interactions were computed using the smooth particle mesh Ewald method. The obtained trajectory was processed utilizing the AMBER11 tool ptraj [26] for RMSD calculations and PMSF for protein and ligand RMSDs. Representative structures of each ligand were chosen from 10,000 trajectory coordinates (last 100 ns) with a k-means clustering algorithm (k = 1) using ptraj. The time course of protein-RMSD (red) and ligand-RMSD (blue) were shown in S2 Fig. Since the RMSD of each model reached a plateau by 100 ns (S2B Fig), the structures of 100 ns were chosen to apply heavy-atom-restrained minimization and they were regarded as an MD-minimized model.

### Synthesis

#### (*S*)-lactisole

**Step 1. Synthesis of methyl (*S*)-2-(4-methoxyphenoxy)propanoate [(*S*)-2a].** To 4-methoxyphenol **1a** (201 mg, 1.62 mmol) in dry CH_2_Cl_2_ (14.9 mL), methyl D-(+)-lactate (253 mg, 2.43 mmol) and PPh_3_ (718 mg, 2.74 mmol) were added at 0 °C. After the reaction mixture was stirred for 10 min at 0 °C, DEAD (486 mg, 2.79 mmol) was slowly added at the same temperature. The reaction mixture was stirred overnight at room temperature and then partitioned between water and CH_2_Cl_2_. The organic layer was washed with brine, dried over MgSO_4_, filtered, and concentrated. The residue was purified by column chromatography (ethyl acetate/n-hexane, 1:9) to yield **(*S*)-2a** (305 mg, 89%). ^1^H NMR (270 MHz, CDCl_3_): *δ* = 6.81 (s, 4H), 4.67 (q, *J* = 6.7 Hz, 1H), 3.73 (s, 6H), 1.58 (d, *J* = 6.9 Hz, 3H) ppm, ^13^C NMR (67.5 MHz, CDCl_3_): *δ* = 172.7, 154.4, 151.5, 116.4, 114.5, 73.4, 55.4, 52.0, 18.4 ppm.

**Step 2. Synthesis of (*S*)-lactisole [(*S*)-3a].** Methyl (*S*)-2-(4-methoxyphenoxy)propanoate **(*S*)-2a** (193 mg, 0.92 mmol) was dissolved in MeOH (17 mL) and H_2_O (2 mL), and then K_2_CO_3_ (399 mg, 2.89 mmol) was added. The reaction mixture was stirred at reflux for 2 hours, cooled to room temperature, and then partitioned between ethyl acetate and water. The water layer was acidified with 1 M HCl aq. and extracted with ethyl acetate. The organic layer was washed with H_2_O and brine and then dried over MgSO_4_, filtered, and concentrated to yield **(S)-3a** (148 mg, 82%). ^1^H NMR (270 MHz, CDCl_3_): *δ* = 6.77 (d, *J* = 3.0 Hz, 4H), 4.62 (q, *J* = 6.9 Hz, 1H), 3.69 (s, 3H), 1.56 (d, *J* = 6.9 Hz, 3H) ppm, ^13^C NMR (67.5 MHz, CDCl_3_): *δ* = 177.8, 154.7, 151.2, 116.7, 114.8, 73.2, 55.7, 18.4 ppm.

#### (*R*)-lactisole

**Step 1. Synthesis of methyl (*R*)-2-(4-methoxyphenoxy)propanoate [(*R*)-2a].** The similar treatment of 4-methoxyphenol **1a** (208 mg, 1.68 mmol) and methyl L-(-)-lactate (262 mg, 2.52 mmol) as that just described gave **(*R*)-2a** (334 mg, 95%). ^1^H NMR (270 MHz, CDCl_3_): *δ* = 6.80 (s, 4H), 4.67 (q, *J* = 6.8 Hz, 1H), 3.72 (s, 6H), 1.58 (d, *J* = 6.9 Hz, 3H) ppm, ^13^C NMR (67.5 MHz, CDCl_3_): *δ* = 172.7, 154.4, 151.5, 116.3, 114.5, 73.4, 55.4, 52.0, 18.4 ppm.

**Step 2. Synthesis of (*R*)-lactisole [(*R*)-3a].** The similar treatment of methyl (*S*)-2-(4-methoxyphenoxy)propanoate **(*S*)-2a** (250 mg, 1.19 mmol) as that just described gave **(*R*)-3a** (209 mg, 89%). ^1^H NMR (270 MHz, CDCl_3_): *δ* = 6.77 (s, 4H), 4.62 (q, *J* = 6.8 Hz, 1H), 3.69 (s, 3H), 1.56 (d, *J* = 6.6 Hz, 3H) ppm, ^13^C NMR (67.5 MHz, CDCl_3_): *δ* = 178.1, 154.6, 151.3, 116.6, 114.7, 73.1, 55.6, 18.4 ppm.

#### (*S*)-2,4-DP

**Step 1. Synthesis of methyl (*S*)-2-(2,4-dichlorophenoxy)propanoate [(*S*)-2b].** The similar treatment of 2,4-dichlorophenol **1b** (338 mg, 2.07 mmol) and methyl D-(+)-lactate (324 mg, 3.11 mmol) as that just described gave **(*S*)-2b** (491 mg, 95%). ^1^H NMR (270 MHz, CDCl_3_): *δ* = 7.30 (d, *J* = 2.6 Hz, 1H), 7.05 (dd, *J* = 8.2, 2.6 Hz, 1H), 6.70 (d, *J* = 8.9 Hz, 1H), 4.65 (q, *J* = 6.8 Hz, 1H), 3.68 (s, 3H), 1.59 (d, *J* = 6.6 Hz, 3H) ppm, ^13^C NMR (67.5 MHz, CDCl_3_): *δ* = 171.7, 152.2, 130.2, 127.5, 127.1, 124.8, 116.1, 74.3, 52.4, 18.4 ppm.

**Step 2. Synthesis of (*S*)-2,4-DP [(*S*)-3b].** The similar treatment of methyl (*S*)-2-(2,4-dichlorophenoxy)propanoate **(*S*)-2b** (397 mg, 1.60 mmol) as that just described gave **(*S*)-3b** (266 mg, 71%). ^1^H NMR (270 MHz, CDCl_3_): *δ* = 10.63 (s, 1H), 7.39 (d, *J* = 2.3 Hz, 1H), 7.16 (dd, *J* = 8.7, 2.5 Hz, 1H), 6.83 (d, *J* = 8.9 Hz, 1H), 4.77 (q, *J* = 6.9 Hz, 1H), 1.72 (d, *J* = 6.9 Hz, 3H) ppm, ^13^C NMR (67.5 MHz, CDCl_3_): *δ* = 176.8, 151.8, 130.4, 127.6, 125.0, 116.5, 74.9, 18.2 ppm.

#### (*R*)-2,4-DP

**Step 1. Synthesis of methyl (*R*)-2-(2,4-dichlorophenoxy)propanoate [(*R*)-2b].** The similar treatment of 2,4-dichlorophenol **1b** (346 mg, 2.12 mmol) and methyl L-(-)-lactate (331 mg, 3.18 mmol) as that just described gave **(*R*)-2b** (524 mg, 99%). ^1^H NMR (270 MHz, CDCl_3_): *δ* = 7.32 (d, *J* = 2.6 Hz, 1H), 7.14 (dd, *J* = 8.7, 2.5 Hz, 1H), 6.78 (d, *J* = 8.9 Hz, 1H), 4.73 (q, *J* = 6.8 Hz, 1H), 3.76 (s, 3H), 1.67 (d, *J* = 6.9 Hz, 3H) ppm, ^13^C NMR (67.5 MHz, CDCl_3_): *δ* = 171.7, 152.2, 130.3, 127.5, 127.1, 124.8, 116.2, 74.4, 52.4, 18.4 ppm.

**Step 2. Synthesis of (*R*)-2,4-DP [(*R*)-3b].** The similar treatment of methyl (*R*)-2-(2,4-dichlorophenoxy)propanoate **(*R*)-3b** (438 mg, 1.76 mmol) as that just described gave **(*R*)-3b** (232 mg, 62%). ^1^H NMR (270 MHz, CDCl_3_): *δ* = 10.97 (s, 1H), 7.31 (d, *J* = 2.6 Hz, 1H), 7.08 (dd, *J* = 8.7, 2.5 Hz, 1H), 6.75 (d, *J* = 8.9 Hz, 1H), 4.69 (q, *J* = 6.8 Hz, 1H), 1.64 (d, *J* = 6.9 Hz, 3H) ppm, ^13^C NMR (67.5 MHz, CDCl_3_): *δ* = 177.1, 151.9, 130.4, 127.6, 127.6, 125.0, 116.4, 73.9, 18.2 ppm.

## Results

### Measurement of the inhibitory activities of (±)-lactisole and (±)-2,4-DP against the human sweet taste receptor with point mutants in T1R3-TMD

Here, we performed a series of cellular experiments on cells stably expressing each point mutant of the human sweet taste receptor to characterize candidate residues in T1R3-TMD that may be involved in the interaction between the inhibitors and the receptor. After the introduction of PCR-based mutations into an expression construct suitable for stable expression of the human sweet taste receptor [9,14,24], we successfully constructed more than 30 cell lines that stably express different receptors, each with a single point mutation in T1R3-TMD (S3 and S4 Figs). To confirm the responsiveness of the cell lines expressing each of the mutant receptors, we first measured the cellular responses to aspartame, an orthosteric agonist that interacts with T1R2-VFTD. Using individual dose-dependent curves, the EC_50_ values for each cell line were calculated, indicating the functionalities of the cell lines used in this study (S1 Table and S3A Fig).

**Fig 1:**
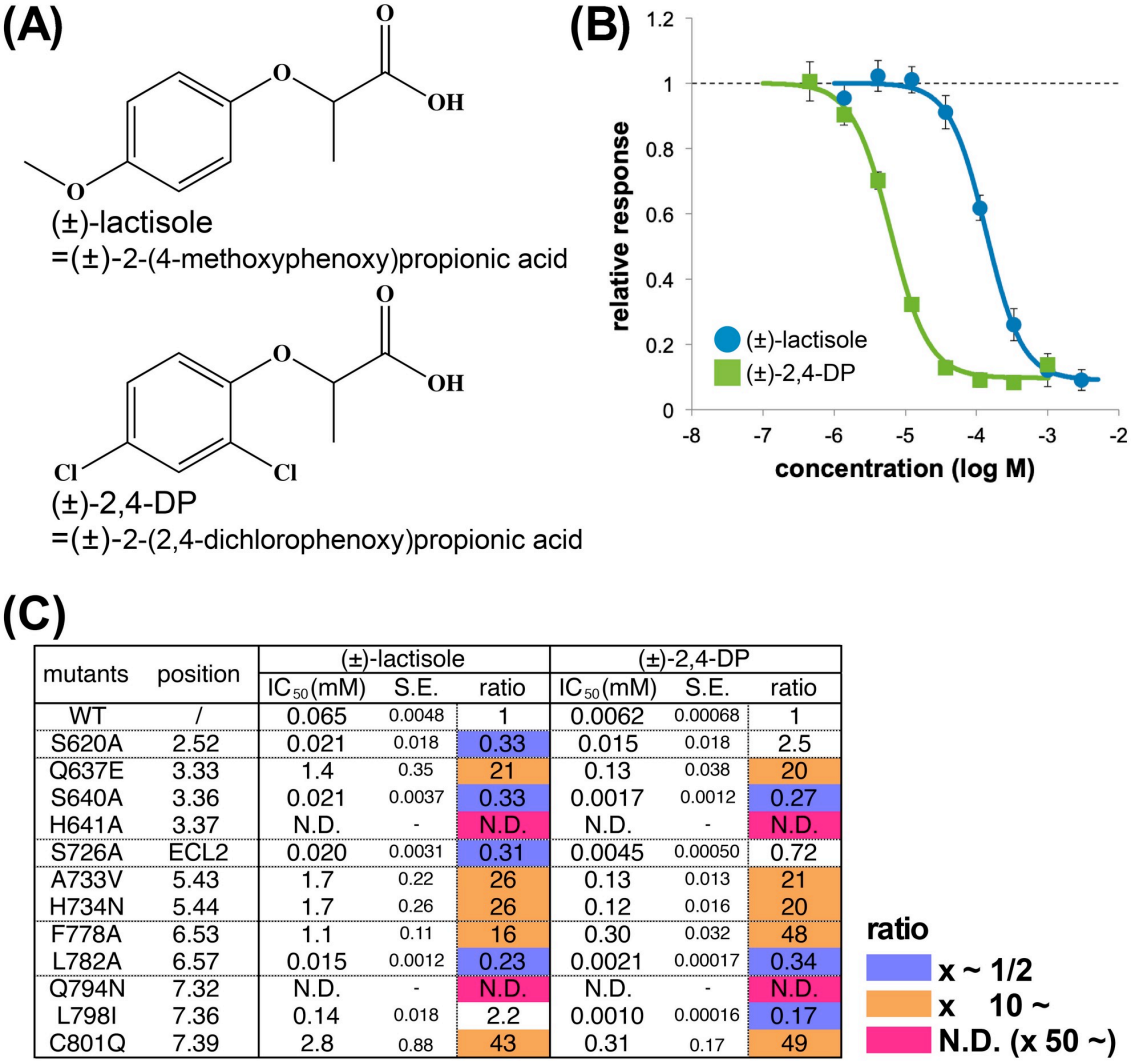
Lactisole and 2,4-DP as sweet taste inhibitors. (A) Structural formulas of (±)-lactisole and (±)-2,4-DP. (B) Dose-response curve showing the inhibition of the cellular responses of cells stably expressing the WT sweet taste receptor by (±)-lactisole and (±)-2,4-DP. Each inhibitor was added to 1 mM aspartame. The results for (±)-lactisole are shown in blue, and those for (±)-2,4-DP are shown in green. Data are shown as the mean ± SEM (n = 4). (C) Summary of mutational analysis for inhibitors (S.E.: standard error of each IC_50_). The results are summarized in S1 Table. Each ratio was calculated by dividing the value for the mutant by that for the WT. Mutants with IC_50_ ratios < 1/2 are colored in blue, ≥ 10 in orange, and ≥ 50 or N.D. in magenta, respectively. N.D., not determined since inhibitory activity was almost entirely eliminated.

Next, we measured the inhibitory activities of (±)-lactisole [(±)-2-(4-methoxyphenoxy)-propionic acid] and (±)-2,4-DP [(±)-2-(2,4-dichlorophenoxy)propionic acid] (Fig 1A) on the cellular responses of the receptor-expressing cell lines in response to aspartame application (S1 Table and S3B Fig). For this purpose, we measured the cellular responses to the application of mixtures containing both 1 mM aspartame and eight different concentrations of inhibitors (Fig 1B, S3B and S5 Figs). The IC_50_ values of the inhibitors for the wild type (WT) receptor-expressing cells were found to be 65 μM for (±)-lactisole and 6.2 μM for (±)-2,4-DP, respectively, confirming that the inhibitory activity of (±)-2,4-DP was approximately 10-fold more potent than that of (±)-lactisole under our experimental conditions (Fig 1B and 1C).

From the results of a series of cellular experiments on all cell lines expressing mutant receptors, the IC_50_ ratio (i.e., IC_50_ for the mutant divided by IC_50_ for the WT) was calculated (S1 Table, also summarized in Fig 1C). Large reductions in the inhibitory activities of both (±)-lactisole and (±)-2,4-DP were confirmed for residues highlighted in orange or magenta, which were therefore considered to be candidates for involvement in the interaction between the inhibitors and the receptor (Fig 1C). Despite the difference in inhibitory potency between (±)-lactisole and (±)-2,4-DP, reductions in the inhibitory activities of both were observed for same seven mutants (Q637E^3.33^, H641A^3.37^, A733V^5.43^, H734N^5.44^, F778A^6.53^, Q794N^7.32^ and C801Q^7.39^) compared to the WT (Fig 1C). Moreover, the H641A^3.37^ and Q794N^7.32^ mutants both nearly abolished the inhibitory activity (S5 Fig). These findings suggested that lactisole and 2,4-DP shared the same binding mode with T1R3-TMD and that H641^3.37^ and Q794^7.32^ in TMD are particularly important residues for interaction with these ligands.

### Simulation of binding modes between lactisole/2,4-DP and the homology model of T1R3-TMD

**Fig 2:**
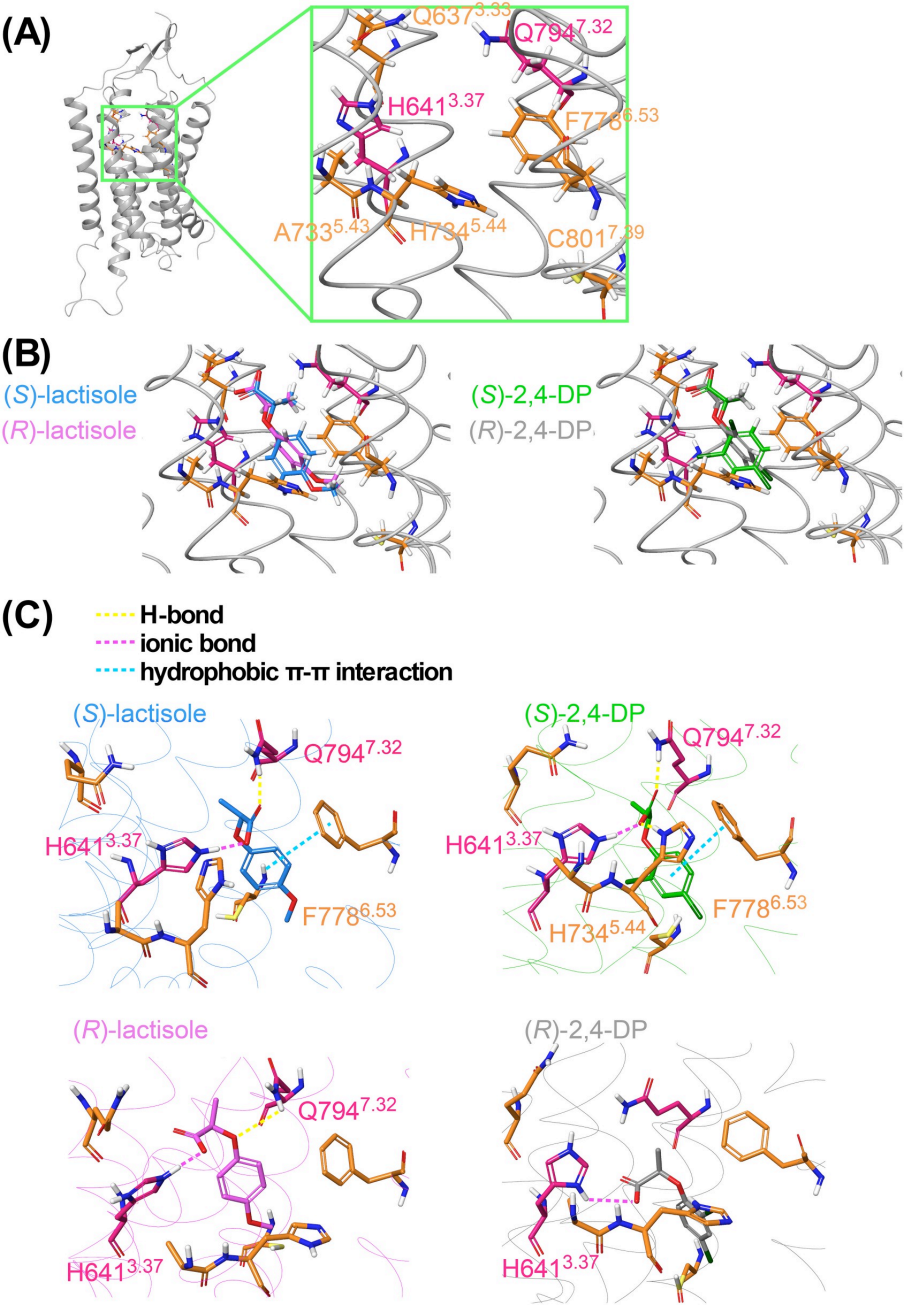
MD Simulations and their pre-simulations for lactisole and 2,4-DP. (A) T1R3-TMD homology model created based on mGluR1 TMD (PDB ID: 4OR2). An enlarged view of the candidate ligand pocket is shown in the right window. Residues extracted by cellular analyses are indicated by colored stick illustrations. (B) Docking modes of (*S*)-lactisole (shown in blue) and (*R*)-lactisole (shown in pink) are shown in the left figure. (*S*)-2,4-DP (green) and (*R*)-2,4-DP (gray) are shown on the right. (C) MD minimizations (100 ns) were performed for each ligand from the docking models. The two left panels show the results for isomers of lactisole (upper panel: (*S*)-lactisole and lower panel: (*R*)-lactisole). The two right panels show the results for isomers of 2,4-DP (upper panel: (*S*)-2,4-DP and lower panel: (*R*)-2,4-DP). The estimated interactions between residues and the ligands are shown by dotted lines in the illustrations after MD: hydrogen bonds are shown in yellow, ionic bonds in magenta, and hydrophobic π-π interactions in cyan.

To explain the result of mutational analyses, we next performed docking simulations and MD-based energy minimizations with the constructed homology model to investigate in detail the differences in the binding configuration between lactisole and 2,4-DP with T1R3-TMD. Prior to MD, a structural homology model of T1R3-TMD was created based on the crystal structure of mGluR1 (PDB ID: 4OR2). A whole view of the homology model and a view around the residues extracted by mutational analyses are shown in Fig 2A. The seven residues are shown by colored stick illustrations. Q637^3.33^, A733^5.43^, H734^5.44^, F778^6.53^ and C801^7.39^ are colored orange, and H641^3.37^ and Q794^7.32^ are colored magenta. All seven residues are positioned near each other in this structural model and seem to constitute a hydrophobic pocket in the upper region of the transmembrane domain. Moreover, the side chains of two residues, H641^3.37^ and Q794^7.32^, which were indicated to be the most important for the interaction between the inhibitors and the receptor, face each other in the model, suggesting that the common chemical structure of the inhibitors would be positioned between the side chains of H641^3.37^ and Q794^7.32^ and that this interaction is critical for their inhibitory activities.

Next, docking simulations of lactisole and 2,4-DP were carried out in the space of a ligand pocket constructed of the seven residues, showing reasonable positions for these ligands. Since both lactisole and 2,4-DP are chiral and since the cellular analysis in this study was carried out with commercially available racemates, both the (*S*)- and (*R*)-isomers of both ligands were simulated (Fig 2B). To verify the orientation of the ligands, we also utilized the information for another ligand, (±)-2-phenoxypropionic acid ((±)-2-PP). 2-PP is a structural analog of lactisole with no methoxy group on its benzene moiety (S1 Table). When the inhibitory activities of (±)-lactisole and (±)-2-PP against each mutant were compared, the results for the C801Q^7.39^ mutant showed a clear difference. Lactisole had less inhibitory activity toward the C801Q^7.39^ mutant, whereas (±)-2-PP had almost the same activity as toward the WT (S1 Table). Since Cys801^7.39^ oriented to the *p*-methoxy group direction of (*S*)-lactisole, the docking results the ligands would be considered to be docked correctly (Fig 2B).

However, because the docking position of each ligand was almost the same, we couldn’t ascertain which parts of particular isomers are important for their inhibitory activities. To identify this, we applied MD simulations with the docking models for 100 ns, and allowed the ligands to converge to the positions where they are energetically stable (Fig 2C). As a result, (*S*)-lactisole had three interactions which comprised ionic bonds with H641^3.37^, F778^6.53^ and Q794^7.32^, whereas (*R*)-lactisole had an ionic interaction with H641^3.37^ and a hydrogen bond with Q794^7.32^ in a different manner than (*S*)-lactisole. Similarly (*S*)-2,4-DP had four interactions with H641^3.37^, H734^5.44^, F778^6.53^ and Q794^7.32^, while (*R*)-2,4-DP only had one ionic interaction with H641^3.37^. In summary, only the (*S*)-isomers of both lactisole and 2,4-DP could maintain the salt bridges between the carboxyl group and H641^3.37^, and also maintain a H-bond between the carboxyl group and Q794^7.32^; in contrast, the (*R*)-isomers could only maintain the interaction between the carboxyl group and H641^3.37^ (Fig 2C). Based on these results, we concluded that the (*S*)-isomers of lactisole and 2,4-DP are predominantly responsible for their inhibitory activities. Further information from 80 ns to 100 ns of MD simulations are shown in S6 Fig. The data show the frequency of each residue’s contact with the ligands during these 20 ns. Because each (*R*)-isomer had more interaction via water compared to (*S*)-isomers, (*S*)-isomers were thought to be more stable during simulations. In particular, 2,4-DP had four specific interactions as shown Fig 2C.

**Fig 3:**
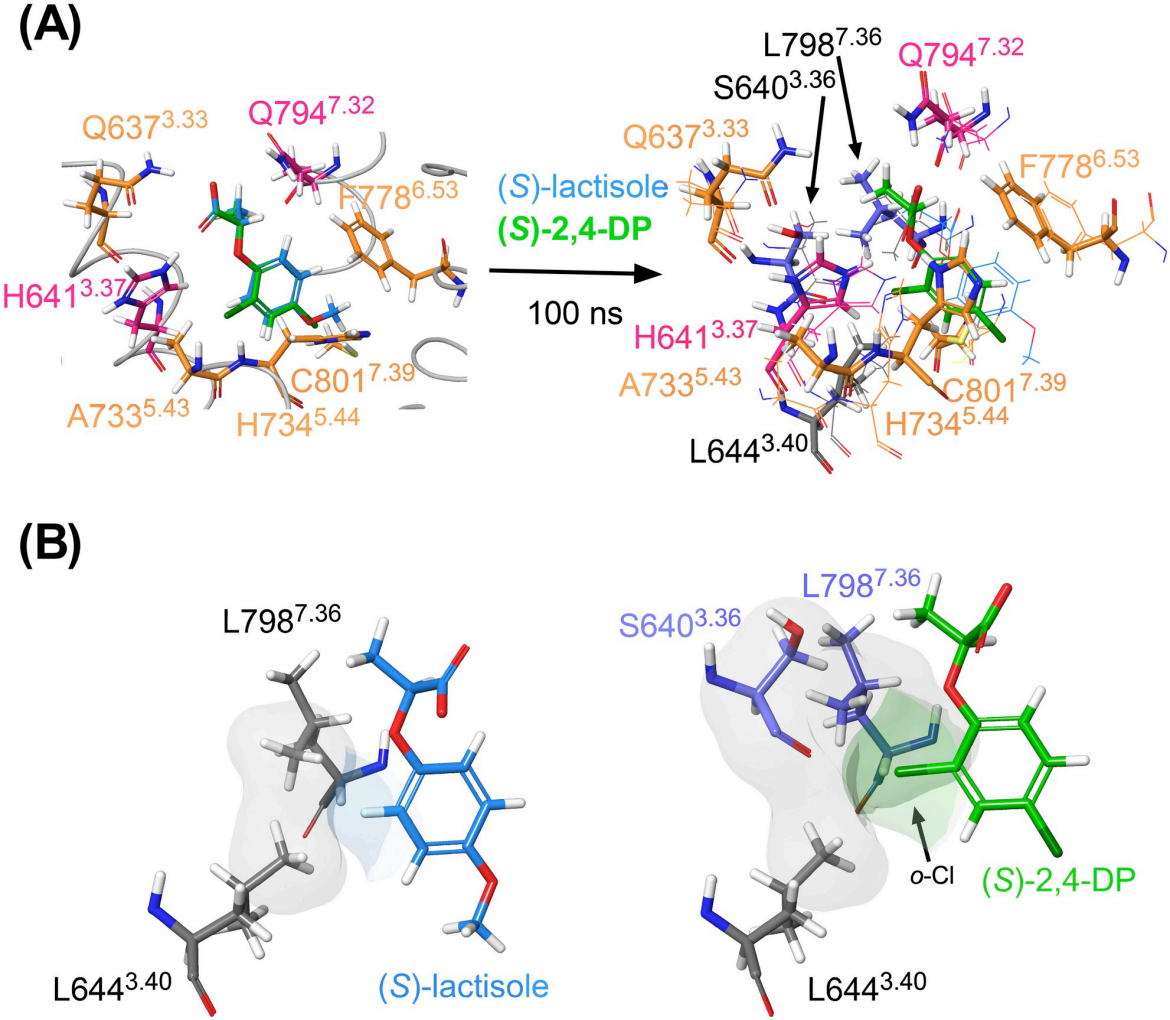
Comparison of the results of MD minimizations for (*S*)-lactisole and (*S*)-2,4-DP. (A) Results of the MD minimization of (*S*)-lactisole (shown in blue) and (*S*)-2,4-DP (shown in green). In the superimposed view after MD minimization, the (*S*)-lactisole model is shown by thin lines, and the (*S*)-2,4-DP model is shown by thick lines. (B) Van der Waals interaction areas between T1R3-TMD and (*S*)-lactisole or (*S*)-2,4-DP are shown.

The results of docking simulations and MD minimizations for (*S*)-lactisole and (*S*)-2,4-DP are superimposed in Fig 3A. Although the docking poses of these compounds were almost identical, the positions of the two ligands were slightly different after 100 ns of simulation (Fig 3A right and S7 Fig). (*S*)-lactisole was closer than (*S*)-2,4-DP to TM7, and correspondingly, (*S*)-2,4-DP was located closer than (*S*)-lactisole to TM3. Moreover, focusing on the *ortho*-position of the aromatic ring of each ligand (Fig 3B), (*S*)-2,4-DP has an *o*-Cl group that engaged in van der Waals interactions with S640^3.36^, L644^3.40^, and L798^7.36^ (Fig 3B, right). In contrast, lactisole has no such functional group and was positioned farther from these residues than 2,4-DP, with interactions that appeared to be weaker (Fig 3B, left). To summarize, the mutational analyses and docking simulations revealed that the same residues were responsible for interaction with lactisole and 2,4-DP; however, the MD minimization revealed a slight difference in position involving the *o*-Cl group of (*S*)-2,4-DP.

To confirm whether (*S*)-lactisole or (*S*)-2,4-DP shifted farther from the appropriate position to act as an inhibitor, we superimposed the (*S*)-2,4-DP minimized model with the crystal structures of mGluR-TMDs (mGluR1 4OR2, mGluR5 4OO9, 5CGC, 5CGD, 6FFI, and 6FFH) [22,23,27,28]. This analysis showed that the position of (*S*)-2,4-DP was similar to the ligands in other receptors (S8 Fig). This result suggested that (*S*)-2,4-DP, but not (*S*)-lactisole, stayed in the correct position to act as an inhibitor and could therefore act as a more potent inhibitor.

### Comparison of the inhibitory activities of the (*S*)-isomer and (*R*)-isomer

**Fig 4:**
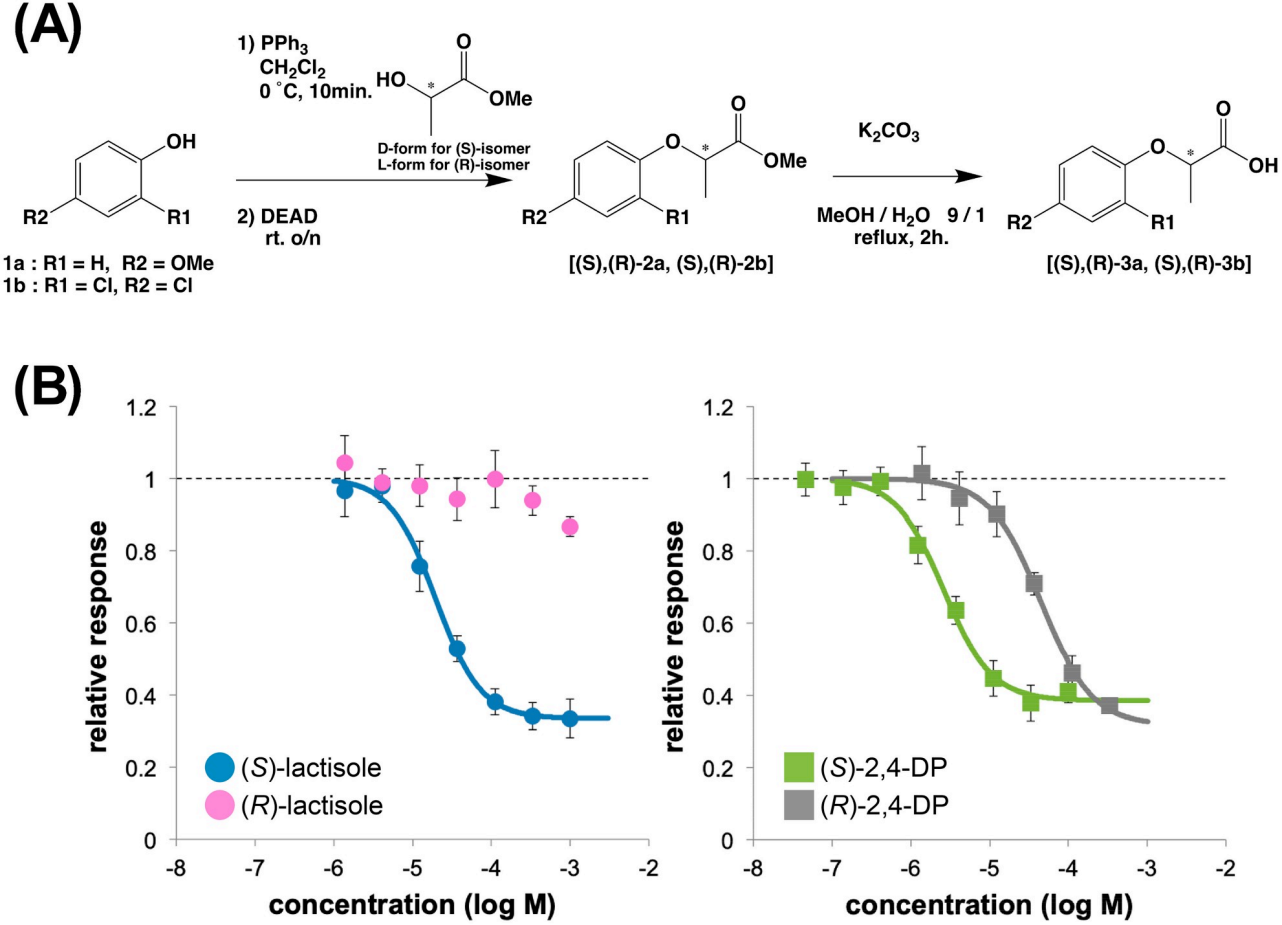
Sweet taste inhibitory activity of (*S*)-, (*R*)-lactisole and (*S*)-, (*R*)-2,4-DP. (A) Flow chart of the synthesis of each isomer of lactisole and 2,4-DP. (B) Cellular response to 1 mM aspartame plus the indicated concentration of each compound against the cells expressing the WT sweet taste receptor. The left panel shows the results for the lactisole isomers: (*S*)-lactisole (blue) and (*R*)-lactisole (pink). The right panel shows the results for the 2,4-DP isomers: (*S*)-2,4-DP (green), and (*R*)-2,4-DP (gray). Data are shown as the mean ± SEM (n = 4).

MD minimization suggested that the (*S*)-isomers of both ligands ((*S*)-lactisole and (*S*)-2,4-DP) interacted with residues of T1R3-TMD. To verify whether this assumption was correct, we synthesized four ligands, (*S*)-lactisole, (*R*)-lactisole, (*S*)-2,4-DP, and (*R*)-2,4-DP, according to the flow chart shown in Fig 4A. The (*S*)-isomers of both lactisole and 2,4-DP showed potent inhibitory activities with the following IC_50_ values: 20 μM for (*S*)-lactisole, 2.6 μM for (*S*)-2,4-DP, and 45 μM for (*R*)-2,4-DP (Fig 4B). (*R*)-2,4-DP was ≥ 10-fold less effective than (*S*)-2,4-DP, and (*R*)-lactisole exerted almost no inhibition at the measured concentration (Fig 4B). This result strongly supports the idea that the (*S*)-isomers interact more strongly with T1R3-TMD, confirming the accuracy of the MD-minimized models constructed in this study.

## Discussion

In this study, we assessed the differences between the interaction modes of lactisole and 2,4-DP using mutational analyses and MD-based energy minimization. Consequently, it was necessary to evaluate the homology model and the validity of the simulation based on comparison between the results of the simulation and experimentally obtained data.

### Homology model

The homology model was created based on the structure of mGluR1-TMD (PDB ID: 4OR2) according to the alignment shown in S1 Fig. Although the alignment was calculated by Prime independently, it was matched with generic GPCR numbers and the same numbered residues of mGluR1 were replaced with residues of T1R3. Because the sequence identity of the TMD between T1R3 and mGluR1 was much higher than that between T1R3 and rhodopsin, this model was thought to be more accurate than that based on rhodopsin. The comparison of these models was also performed by Chéron *et al*. [29].

Seeing the model, all seven residues identified in our mutational analysis (Fig 1C) were located near one another (Fig 2A). Furthermore, because H641^3.37^ and Q794^7.32^, the two most important residues, were facing each other, these two residues are strongly suggested to interact with inhibitors (Fig 2A). Four residues out of the seven, Q637^3.33^, H641^3.37^, A733^5.43^ and F778^6.53^, were previously reported as residues that interacted with lactisole [11], whereas H734^5.44^, Q794^7.32^ and C801^7.39^ were newly discovered in this study. A previous study reported that R723^45.51^ interacts with the carboxyl group of lactisole [11], whereas in our hands, the IC_50_ of R723A^45.51^ did not differ significantly relative to the WT receptor (S1 Table). This difference could be explained in at least two ways. First, in the previous study, each mutant was measured at only three concentrations, preventing the influence of lactisole in R723A^45.51^ from being accurately evaluated. In contrast, in this study, the response of each mutant was measured at eight inhibitor concentrations, and the IC_50_ values obtained should be more accurate. The second possibility involves Q794^7.32^, another important residue. In a previous study, the Q794A^7.32^ mutant was reported to be nonfunctional [11]. Based on this observation, we created the new Q794N^7.32^ mutant, revealing that Q794^7.32^ was an important residue for interaction with lactisole and 2,4-DP (S5 Fig).

### Ligand docking

H641^3.37^ and Q794^7.32^, which face each other, were considered to be the most important residues for the binding of both lactisole and 2,4-DP (Fig 3A). In both docking models, these residues formed salt bridges via the carboxyl groups of the ligands, and the docking poses are very reasonable. In addition, regarding the C801Q^7.39^ mutation, C801^7.39^ lies in the deep part of the ligand pocket (Fig 2A). In this study, we replaced this residue with a bulkier residue, Gln, to fill the space where the ligand should interact. Indeed, the inhibitory activity of both (±)-lactisole and (±)-2,4-DP, was greatly reduced by this mutation (Fig 1C). On the other hand, the IC_50_ of (±)-2-PP, in which the *p*-position methoxy group was removed from lactisole, was not crucially affected by the C801Q^7.39^ mutation (S1 Table and S5 Fig). Based on these findings, we expected the *p*-position groups of lactisole and 2,4-DP to be oriented toward the deep part of the ligand pocket (Fig 2B).

### MD-based energy minimization

The simulation results differed greatly between the (*S*)- and (*R*)-isomers for both lactisole and 2,4-DP, and both ligands exhibited stronger receptor-ligand interactions in the (*S*)-isomer-minimized models (Fig 2C). To confirm this result, we synthesized all four isomers and compared their inhibitory activities (Fig 4). Given that the inhibitory activities of each isomer were consistent with the simulation results, we concluded that the results strongly supported the accuracy of the MD-minimized models in this study.

Furthermore, we compared some ligands in crystal structures of class C GPCRs (PDB ID: 4OR2 (mGluR1), and 4OO9, 5CGC, 5CGD, 6FFH and 6FFI (mGluR5)) with the (*S*)-2,4-DP-minimized model (S8 Fig). The residues involved in receptor-ligand interactions, such as those at positions 5.44, 6.53, and 7.39, were present at the same position. This would indicate that MD minimization was performed appropriately. In addition, these residues are very important for ligands to act as NAMs in class C GPCRs [22,23,27,28]. The interaction with T815^7.32^ was also important for the ligand FITM to function as an NAM with mGluR1 [23]. Similarly, the 7.32 position in T1R3, Q794^7.32^, is important for the function of 2,4-DP as an NAM with T1R3 (Fig 1C). Based on these observations, we conclude that the results of our simulation would be reasonable.

Next, we examined the commonalities and differences between the inhibitory mode of action of lactisole and 2,4-DP. Both ligands have lactic acid skeletons (Fig 1A), and their (*S*)-isomers interact more strongly with T1R3-TMD (Fig 2B and 2C). Moreover, both ligands interact with the same residues (Fig 3A). This result might be related to the fact that lactisole is found in coffee beans primarily as the (*S*)-isomer [20].

In contrast, one difference between the two compounds involved the behaviors of the (*R*)-isomers. (*R*)-2,4-DP had some inhibitory activity, whereas (*R*)-lactisole had barely any (Fig 4B). Thus, the inhibitory activity of lactisole was dependent mainly on the carboxyl group, whereas 2,4-DP interacted with T1R3-TMD via two moieties, the carboxyl group and the aromatic ring with two Cl groups. The MD-minimized results of (*S*)-lactisole and (*S*)-2,4-DP (Fig 3A) show clearly that (*S*)-lactisole was closer to TM7 than 2,4-DP. Thus, while (*S*)-lactisole was stabilized in the vertical dimension of the ligand pocket via its carboxyl group, there was not sufficient interaction in the horizontal dimension. In contrast, in (*S*)-2,4-DP, the *o*-position Cl group engaged in hydrophobic interactions with S640^3.36^, L644^3.40^, and L798^7.36^ in the horizontal dimension (Fig 3B). In addition, in the mutational analysis, only (±)-2,4-DP had a lower IC_50_ for the L798I^7.36^ mutant (Fig 1C). This result provides further evidence that the *o*-position Cl group is involved in the interaction with L798^7.36^.

Based on the above, we concluded that the difference in potency between (*S*)-lactisole and (*S*)-2,4-DP was governed by their stability in the ligand pocket of T1R3-TMD: both ligands were stabilized in the vertical dimension by the common 2-phenoxypropionic acid skeleton, but only (*S*)-2,4-DP was stabilized in the horizontal dimension via its *o*-Cl group (Fig 3A and 3B).

In the experiments described above, we succeeded in clarifying the difference between lactisole and 2,4-DP using interaction models (Fig 3). However, since these models were only simulated using low-molecular weight NAMs, it is difficult to explain the interaction mechanism of larger NAMs such as gymnemic acid [13]. Indeed, three residues in T1R3-TMD were reported to be quite important for gymnemic acids: H641^3.37^, A733^5.43^, and R725^ECL2^ in extracellular loop 2 (ECL2) [13]. Thus, although H641^3.37^and A733^5.43^ were commonly important for binding of both lactisole and 2,4-DP, gymnemic acid has another interaction mode extending from the ligand pocket to the vicinity of ECL2.

At any rate, it may be possible to propose a design for further high-affinity ligands using the minimized model created in this study. In concrete terms, comparison of the ligand positions in the mGluR crystal structures with those of (*S*)-2,4-DP in S8 Fig revealed that the other mGluR ligands were extended in the direction of the two Cl groups in 2,4-DP. On the other hand, in the L798I^7.36^ mutant, 2,4-DP was a more potent inhibitor (Fig 1C), and there was space for (*S*)-lactisole to slide away (Fig 3B). These data showed that a more potent sweet taste inhibitor could be created by designing a derivative with larger hydrophobic functional groups in both the *o*- and *p*-directions of (*S*)-2,4-DP.

## Supporting information

S1 Table Summary of the results of cellular experimental analyses for each cell line that stably expressed the wild-type (WT) or a point mutant of the human sweet taste receptor. The EC_50_ values for aspartame and the IC_50_ values for (±)-lactisole, (±)-2,4-DP, and (±)-2-PP (2-phenoxypropionic acid) are indicated (S.E.: standard error of each EC_50_ or IC_50_). Each ratio was calculated by dividing the value for the mutant by that for the WT. Mutants with ratios < 1/2 are colored in blue, ≥ 5 in gray, ≥ 10 in orange, and ≥ 50 or N.D. in magenta, respectively. N.D., not determined since the inhibitory activity was almost completely eliminated. References upon which we created mutations are indicated by superscripts. Some of them were modified to other mutations because they had been reported as inactive or hyperactive mutations.
(TIF)

S1 Fig The Alignment of mGluR1, 5 and T1R1, 2 and 3. (A)The alignment of the TMD regions of five receptors: mGluR1, mGluR5, T1R1, T1R2 and T1R3. Each area surrounded by a green line indicates transmembrane (TM) regions. (B) Sequence identities of each receptor are shown in the upper right of the table, while sequence similarities of each receptor are shown in the lower left of the table. It should be noted that rhodopsin and β2-adrenoceptor (β2-AR) are categorized as class A GPCRs.
(TIF)

S2 Fig Time course plots of protein-RMSD and ligand-RMSD. (A) Each RMSD of four MD simulations is shown. Protein RMSD is shown in blue, and ligand RMSD is shown in red. Upper left: is the one of (*S*)-lactisole. Lower left: (*R*)-lactisole. Upper right: (*S*)-2,4-DP. Lower right: (*R*)-2,4-DP. (B) The time course during 80 ns to 100 ns.
(TIF)

S3 Fig Plots of cellular experimental analyses for each cell line that stably expressed the wild-type (WT) or a point mutant of the human sweet taste receptor. (A) Dose response curves against aspartame of the all utilized mutants. Data are shown as the mean ± SEM (n = 3–4). (B) Dose response curves against 1 mM aspartame and each concentration of lactisole. Data are expressed as the mean ± SEM (n = 4).
(TIF)

S4 Fig Snake plot of T1R3-TMD. Residues mutated in this study are highlighted. Residues thought to interact with both lactisole and 2,4-DP, based on the cellular analyses in this study, are colored in orange and magenta.
(TIF)

S5 Fig Sweet taste inhibitory activity of three ligands in each mutant in which the inhibitory activities were largely reduced. Dose-response curves of WT and seven mutant receptors for three ligands are shown: (±)-lactisole (blue), (±)-2,4-DP (green), and (±)-2-PP (red). Data are shown as the mean ± SEM (n = 4).
(TIF)

S6 Fig The residue-ligand contact frequencies during MD-simulations. The contact frequency during 80 ns– 100 ns is shown. Hydrophilic interactions are shown in violet and hydrophobic interactions are shown in green. The percentages shown on line denote contact frequency: contacts with >50% frequency are shown in Fig 2C. The thick lines surrounding each ligand indicate the type of adjacent molecule: Blue indicates hydrophilic molecules including water and green indicates hydrophobic molecules.
(TIF)

S7 Fig Comparison of pre- and post-MD simulations. Superimposed view of docking-model and MD-simulated model. The docking model is shown in thin lines and the MD-simulated model is shown in thick lines. (A): (*S*)-lactisole model. (B): (*S*)-2,4-DP model.
(TIF)

S8 Fig Superimposed view of (*S*)-2,4-DP model and other class C GPCR structures. Comparison of the (*S*)-2,4-DP minimized model and crystal structure of other class C GPCRs. MD-minimized models for (*S*)-2,4-DP (green) and six other ligands (FITM from 4OR2, shown in light blue; mavoglurant from 4OO9, shown in orange; Compound14 from 5CGC, shown in violet; HTL14242 (Compound25) from 5CGD, shown in magenta; MMPEP from 6FFI, shown in dark gray, and fenobam from 6FFH, shown in light gray) are superimposed.
(TIF)

S1 Appendix The models created in this study. Four models are available by downloading S1 Appendix.zip. This file contains (S)-lactisole_dock.pdb, (S)-lactisole_MD.pdb, (S)-2,4-DP_dock.pdb, and (S)-2,4-DP_MD.pdb.
(ZIP)
